# Exosomal SOX21-AS1 Regulates EREG by Sponging miR-451a and Promotes the Malignancy of Pancreatic Ductal Adenocarcinoma

**DOI:** 10.7150/jca.95014

**Published:** 2024-04-23

**Authors:** Yong Yan, Jinyi Wang, Bin Xu, Jianming Ni, Tu Dai, Liying Wang, Hao Wang, Zhiyuan Hua, Kuan Li, Yongping Zhou

**Affiliations:** 1 Department of Hepatobiliary Surgery, The Affiliated Wuxi No. 2 People's Hospital of Nanjing Medical University, Wuxi, China.; 2 Hepatobiliary Center, The First Affiliated Hospital of Nanjing Medical University, Nanjing, China.; 3 Department of Radiology, The Affiliated Wuxi No. 2 People's Hospital of Nanjing Medical University, Wuxi, China.; 4 Department of Hepatobiliary Surgery, Jiangnan University Medical Center, JUMC, Wuxi, China.; 5 Department of Hepatobiliary Surgery, Kunshan Hospital of Traditional Chinese Medicine, Kunshan, China.

**Keywords:** PDAC, SOX21-AS1, exosome, miR-451a, EREG, angiogenesis

## Abstract

The incidence and mortality of pancreatic ductal adenocarcinoma (PDAC) have increased. Exosomes, as a regulatory mode of intercellular communication, contain lncRNAs. SOX21-AS1 has been studied in other cancers, and its expression is elevated in PDAC, but its role in PDAC remains unclear. First, we analyzed the expression of lncRNAs in PDAC tissues and nontumor tissues through the TCGA database. Next, the results of the RT-qPCR experiment confirmed the prediction that the expression of SOX21-AS1 was elevated in PDAC tissues. *In vivo* and *in vitro* cell function assays confirmed that the degree of malignancy of PDAC was proportional to the expression of SOX21-AS1. In addition, through exosome isolation and uptake experiments, we first found that PDAC could secrete exosomal SOX21-AS1 and play an angiogenic role in HUVECs. Subsequently, the relationship between SOX21-AS1, miR-451a and epiregulin (EREG) was verified through database prediction and analysis and RIP assays. Finally, functional recovery assays *in vivo* and *in vitro* verified that SOX21-AS1 regulates the expression of EREG through combination with miR-451a and thus promotes the malignancy of PDAC. SOX21-AS1 was upregulated in PDAC. The upregulation of SOX21-AS1 can stimulate the proliferation, migration, invasion, stemness and epithelial-mesenchymal transition (EMT) progression of PDAC cells. Furthermore, PDAC cells secrete exosomal SOX21-AS1, which is absorbed by HUVECs and promotes angiogenesis. Our study first identified that SOX21-AS1 promotes the malignancy of PDAC through the SOX21-AS1/miR-451a/EREG axis, and also that exosomal SOX21-AS1 promotes angiogenesis in PDAC.

## Introduction

PDAC is the most aggressive malignant tumor. The incidence of PDAC is increasing, and the 5-year survival rate is less than 10% 1-3. Gemcitabine-based chemotherapy is still ineffective, with a 3-year recurrence rate of more than 60% and an overall survival of approximately 10 months 4, 5. The excessive abundance of extracellular matrix in the tumor microenvironment leads to abnormalities in cellular physiological mechanisms, high interstitial pressure, vascular collapse and low nutrition, which make it difficult for cytotoxic chemotherapy drugs to penetrate and concentrate in tumor tissues. Simultaneously, chemotherapeutic resistance greatly reduces the sensitivity of tumors to chemotherapeutic agents 6-8. Therefore, research on the underlying molecular mechanisms of the malignancy of PDAC and the search for new early biomarkers have become frontier topics and research hotspots in this field.

Long noncoding RNAs (lncRNAs) are a class of RNAs that are more than 200 nucleotides in length and have little or no protein-coding potential; they are important RNA molecules involved in a variety of biological functions 9. Although the function of most lncRNAs remains to be elucidated, some lncRNAs have been indicated to be related to human diseases such as cancer 10. The subcellular localization of lncRNAs determines the function and molecular mechanism of lncRNAs 11. lncRNAs located in the cytoplasm can interfere with protein-modified translation and influence gene expression by acting as decoys for miRNAs and proteins 12. The lncRNA SOX21 antisense divergent transcript 1 (SOX21-AS1) is a transcript of 2986 nt located on chromosome 13q32.1^13^. Research has demonstrated that SOX21-AS1 plays an important regulatory role in the development of multifarious cancers. For example, SOX21-AS1 can act as an oncogene by partially inhibiting P57 in lung cancer 14-16. SOX21-AS1 affects the progression of hepatocellular carcinoma by epigenetically silencing P21 16. The TCGA database data show that the expression of SOX21-AS1 is upregulated in PDAC, is associated with poor prognosis and may interact with miR-451a and EREG. However, the role of SOX21-AS1 in PDAC and its molecular mechanism remains unclear.

In recent years, various lines of evidence have indicated that extracellular vesicles (EVs) are essential signal transduction factors in intercellular communication 17. Exosomes (EXs) are extracellular vesicles with a double membrane structure that are 30 to 150 nm in diameter and are characterized by high stability in extracellular fluid and circulation 18, 19. Exosomes take up a large number of bioactive molecules, including proteins, RNA, DNA, cholesterol, and neuropeptides, which enables them to fulfil an exchange function between tumor and nontumor cells 20. Studies have proven that the lncRNAs contained in exosomes secreted by cancer cells can be delivered to recipient cells and regulate the RNA expression of those cells 21. For example, lincRNA-ROR carried by exosomes can influence the sensitivity of hepatocellular carcinoma cells to chemotherapy 22. lncRNAs are also key mediators involved in intercellular signal transduction. We found that SOX21-AS1 promotes the proliferation, migration, invasion and stemness of PDAC. In addition, SOX21-AS1 in exosomes secreted by PDAC cells can be taken up by HUVECs and promotes angiogenesis. Moreover, we confirmed that the SOX21-AS1/miR-451a/EREG axis is a novel regulatory mechanism associated with the malignancy of PDAC, providing a new therapeutic target for PDAC therapy.

The lncRNA/miRNA/mRNA network in PDAC was analyzed by bioinformatics database. Our study aims to elucidate the regulatory role and mechanism of SOX21-AS1 in PDAC. Research on the pathogenesis and development of PDAC can further understand the etiology and progression of PDAC, and also provide new methods and ideas for the diagnosis and treatment of PDAC in the future.

## Materials and methods

### Sample collection

Specimens were collected as previously described by Nie *et al.* 23. This study was approved by the Research Ethics Committee. Inclusion criteria: (1) Specimens were confirmed to be pancreatic ductal adenocarcinoma (PDAC) by histopathological examination; (2) 18 years old to 85 years old; (3) Eastern Cooperative Oncology Group (ECOG) score of 0-2; (4) Patients or authorized relatives of the patients signed the informed consent before enrolment. Exclusion criteria: (1) patients who had received radiotherapy, chemotherapy or other related antitumor therapies before surgery; (2) history of other tumors; (3) patients in pregnancy or lactation; (4) combined organ failure or other serious diseases;(5) combined neurological or psychiatric history.

Eighty isolated PDAC specimens and corresponding adjacent normal tissue specimens were frozen in liquid nitrogen for subsequent study. All specimens were confirmed by pathologists as PDAC and graded based on pathology.

### Cell line maintenance and transfection

The normal human pancreatic ductal cell line HPDE and pancreatic adenocarcinoma cell lines CFPAC-1, AsPC-1, BxPc-3, PANC-1 and MIA PaCa-2 were purchased from the American Type Culture Collection (ATCC, Manassas, VA) and incubated in high glucose Dulbecco's Modified Eagle Medium (DMEM) (Invitrogen, Thermo Fisher Scientific, Inc., Carlsbad, CA, USA) supplemented with 1% penicillin/streptomycin (Invitrogen), 10% foetal bovine serum (FBS; Gibco, Life Technologies, Carlsbad, CA, USA) and 25 mmol/L HEPES (Thermo Fisher, Dreieich, Germany) at 37 °C in a humidified atmosphere with 5% CO2. HUVECs (Institute of Cell Biology of the Chinese Academy of Sciences, Shanghai, China) were cultured in MCDB 131 medium (GIBCO) supplemented with 5% microvascular growth supplement (GIBCO) and 1% GlutaMAX (GIBCO) under the same conditions as those used for PDAC 24, 25. Lentiviruses overexpressing SOX21-AS1 (SOX21-AS1), a small hairpin RNA (shRNA) against SOX21-AS1 (shSOX21-AS1), miR mimic, miR inhibitor, EREG overexpression vector (EREG), shRNA targeting EREG (shEREG) and the corresponding NCs (NC, shNC, mimic NC, inhibitor NC, EREG NC and shEREG NC) were procured from GenePharma (Shanghai, China). Puromycin was used to screen the transfected cells 25.

### RNA acquisition and real-time quantitative PCR (RT-qPCR)

Total RNA was extracted from clinical samples and cultured cell lines using the TRIzol kit (Invitrogen, CA, USA). Total RNA was then reverse transcribed into complementary DNA (cDNA) using gene‐specific primers synthesized by Ribo Biotech (Guangzhou, Guangdong, China). All samples were amplified in 384-well plates according to the instructions. Differences in fold expression were calculated in Excel (Microsoft Corporation, Redmond, WA, USA) using the 2^-ΔΔCt^ method. Genes and primers were listed in Table S1.

### Fluorescence in situ hybridization (FISH)

FISH was used to determine the subcellular localization of SOX21-AS1 in PDAC. The SOX21-AS1 probe, 18S probe and U6 probe were synthesized by (RiboBio, Guangzhou, China) FISH experiments were conducted using a fluorescent in situ hybridization kit (RiboBio, Guangzhou, China) 26. Briefly, logarithmic growth cells were seeded on confocal dishes at 5×10^3^ cells per well. After fixation, the membrane was lysed with 0.5% Triton X-100, and the FISH probe was diluted (1:50) before being added to the petri dish and cultured overnight at 37 °C. After hybridization, the cells were stained with DAPI-Antifade for 10 min, and the cell localization map was visualised by confocal microscopy.

### Cell counting kit-8 (CCK-8) analysis

The transfected cells were inoculated at a density of 5×10^3^ cells/well in 96-well plates, and 6 replicate wells were used for each group. The medium and CCK-8 solution (Dojindo Laboratories, Kumamoto, Japan) were configured in a ratio of 9:1. Next, 100 μl CCK-8 solution was added to each well and cultured for 2 h at different times (0, 24, 48, and 72 h). The absorbance at 450 nm was measured using a microplate reader (Thermo Scientific, USA) as previously reported 27. The growth curve was drawn based on the test results.

### Colony formation assay

PDAC cells in the logarithmic phase were counted to measure viability. The transfected cells were inoculated in 6-well plates (5×10^2^ cells/well) and cultured for 14 d, during which the medium was changed every 3d. The cells were removed from the incubator, fixed with 4% paraformaldehyde for 30 min, and stained with a crystal violet staining solution (Beyotime, Shanghai, China) for 30 min. After dyeing, the dye was dried naturally and photographed, and the results were analyzed. Data collected from three independent experiments are expressed as the mean colony count ± SD.

### 5-Ethynyl-20-deoxyuridine (EdU) proliferation assay

EdU staining was performed using an EdU staining proliferation kit (RiboBio, Guangzhou, China). The transfected cells were inoculated in 24-well plates at a density of 1×10^5^ cells/well. The cells were then incubated with an EdU reaction mixture (provided with the kit) for 2 h, followed by DAPI and Apollo staining according to standard laboratory protocol. After staining, the cells were observed under a fluorescence microscope at a magnification of 200x, and the number of EdU-positive cells was counted in 5 randomly selected fields.

### Scratch wound healing assay

The migration of cells was evaluated by scratch wound healing assay, as mentioned above 28. PDAC cells were cultured in 6-well plates at a density of 2×10^5^ cells/well. When the cell density reached approximately 90%, straight lines were drawn on the surface of the cultured holes. Cell migration was observed under a microscope at 0 and 48 h, and photographs were taken. Finally, the gap distance was quantified and analysed using ImageJ software.

### Transwell assay

PDAC cells (2×10^4^ migrating cells, 4 × 10^4^ invading cells) were placed in the upper compartment of the transwell chamber (precoated with substrate for the invasion test) using 300 μl serum-free medium. Next, 500 μl of complete medium containing 10% FBS was added to the lower chamber. After 48 h of culture, the cells on the cell membrane were fixed with methanol for 20 min and stained with a 0.1% crystal violet solution for 30 min. Migrated and invaded cells were observed and photographed under a microscope.

### Sphere formation

The transfected cells (5×10^2^ cells/well) were seeded into a low-attachment culturing 6-well plate (Corning, USA) and cultured in a medium mixture containing DMEM/F12 (HyClone, USA), methylcellulose (Sigma), B27 (Sigma), bFGF (20 ng/mL, Sigma), and EGF (10 ng/mL, Sigma). The cells were incubated for 14 d, and the medium was changed every 4 d. The cells were recorded and photographed, and the balls formed were counted using ImageJ software.

### Western blotting

Phenylmethylsulfonyl fluoride (PMSF) and radioimmunoprecipitation (RIPA) buffer were prepared at a ratio of 1:100 for the extraction of total protein from cell lines and tissues. The protein was isolated by 10% sodium dodecyl sulfate-polyacrylamide gel electrophoresis (SDS-PAGE) and then transferred to a polyvinylidene fluoride (PVDF) membrane. After blocking, the PVDF films were successively incubated in primary antibody and secondary antibody. Finally, exposure was performed, and the grey value was analyzed using ImageJ software. Antibodies are listed in Table S2.

### Subcutaneous tumor model

The Ethics Committee Review Board approved the animal experimentation program, which strictly followed the "Guidelines for The Care and Use of Laboratory Animals" and the ethical guidelines for animal experiment institutions. Cells transfected with lentivirus were centrifuged and suspended in normal saline at a concentration of 1×10^8^ cells/ml. Four-week-old female BALB/C nude mice were selected, with 5 mice in each group. Each mouse was subcutaneously injected with 100 µl of cell suspension under the armpit of the left forelimb, and the time and the name of the injected cell sample were recorded. Subcutaneous tumor volume was recorded every 3 d, and the formula for calculating the volume was volume = 1/2× length × width^2^. After 28 d, the mice were euthanized. Subcutaneous tumors were removed and photographed, and their volume and mass were calculated. Some of the cells were stored in formaldehyde for subsequent immunohistochemistry, and some of the cells were frozen at -80 °C for protein and RNA extraction.

### Immunohistochemistry (IHC)

Immunohistochemical methods were used to quantify the expressions of Ki-67, VEGF, E-cadherin and Vimentin in the subcutaneous tumor tissues of nude mice from different treatments. After isolation, the samples were fixed with formaldehyde, embedded with paraffin, and the tissues were then sectioned. After antigen recovery, the slides were incubated with primary antibody overnight. After incubation with secondary antibody, immunohistochemical staining was performed with diaminobenzidine (DAB). The samples were then subjected to haematoxylin reverse staining, dehydration, and sealing. Antibodies are listed in Table S2. All images were taken with a light microscope 29.

### Isolation and identification of EXs

EX-depleted FBS was obtained by ultracentrifugation at 120,000 g at 4 °C, followed by filtration through 0.22-μm filters according to the manufacturer's instructions. PANC-1 cells were cultured in DMEM containing 10% EX-depleted FBS. EXs were isolated with ExoQuick reagent (System Biosciences, Palo Alto, CA, USA). The precipitated EXs were resuspended in 100 μL PBS and stored at -80 °C. The surface morphology and ultrastructure of exosomes in the supernatant of PANC-1 cells were analyzed by transmission electron microscopy (TEM). Nanoparticle tracking analysis (NTA) was performed using a Zetaview tracking analyzer (Particle Metrix) equipped with a fast video capture function to calculate the exosome size distribution.

### EX uptake

Based on the manufacturer's instructions, EXs (20 μg) were labelled with PKH67 green fluorescent dye (Sigma-Aldrich) 3. Labelled EXs were resuspended and added to unstained HUVECs for EX uptake experiments. Images were captured using a confocal microscope (Zeiss Meta 510, Thornwood, NY, USA) after 12 h of incubation at 37 °C.

### Tube formation assay

First, Matrigel matrix (Corning, #356231) was melted in a 4 °C refrigerator. Next, 300 µl Matrigel matrix was added to each well of the 24-well plate and then placed into an incubator for 1 h until solidification of the Matrigel matrix. Finally, 1×10^5^ HUVECs were placed into each well and incubated for 8 h. The cell morphology was observed under a microscope and photographed 30.

### Chicken chorioallantoic membrane assay (CAM)

One hundred chicken embryos were incubated at 37.5 °C±0.5 °C for 3 d. The viability of the chicken embryos was confirmed by candling. A total of 95 well-developed chicken embryos were obtained. On the 7th day of incubation, the surface was first disinfected with 75% ethanol, and then a 1.0 cm×1.0 cm window was created in the air chamber. The treated HUVECs were injected into the chicken embryos (23/group), and filter paper was then fixed over the chicken embryos with tape. The tape covering the chicken embryo was later removed and the embryo was fixed with formaldehyde and photographed 31.

### RNA binding protein immunoprecipitation (RIP) assay

The RIP experiment was performed using a Magna RIP immunoprecipitation kit (Merck Millibo, Billerica, Massachusetts, USA). After the PDAC cells were lysed, the supernatant was collected and antibody was added and incubated overnight. Next, protein A/G magnetic beads (Abcam Inc., Cambridge, MA, USA) were added and incubated again for 1 h. Finally, the results were verified by RT-qPCR 32.

### Dual-luciferase reporter assay

The pGL/Luc-SOX21-AS1-WT/pGL/Luc-SOX21-AS1-MUT and pGL/Luc-EREG-WT/pGL/Luc-EREG-MUT vectors (Ambion, Austin, TX, USA) were constructed and then cotransfected with hsa-miR-451a mimics, inhibitors or miR-NC into CFPAC-1 and PANC-1 cells using Lipofectamine 3000 (Invitrogen). The transfected cells were lysed to detect dual-luciferase activity 33.

### Statistical analysis

Statistical analysis and graphing of the data were performed using GraphPad Prism 7.0 (GraphPad) and SPSS version 18.0. The data are presented as the mean ± standard deviation of three independent experiments. P < 0.05 indicates statistically significant differences.

## Results

### SOX21-AS1 expression is upregulated in PDAC tissues and cell lines and correlates with poor prognosis

**Figure 1:**
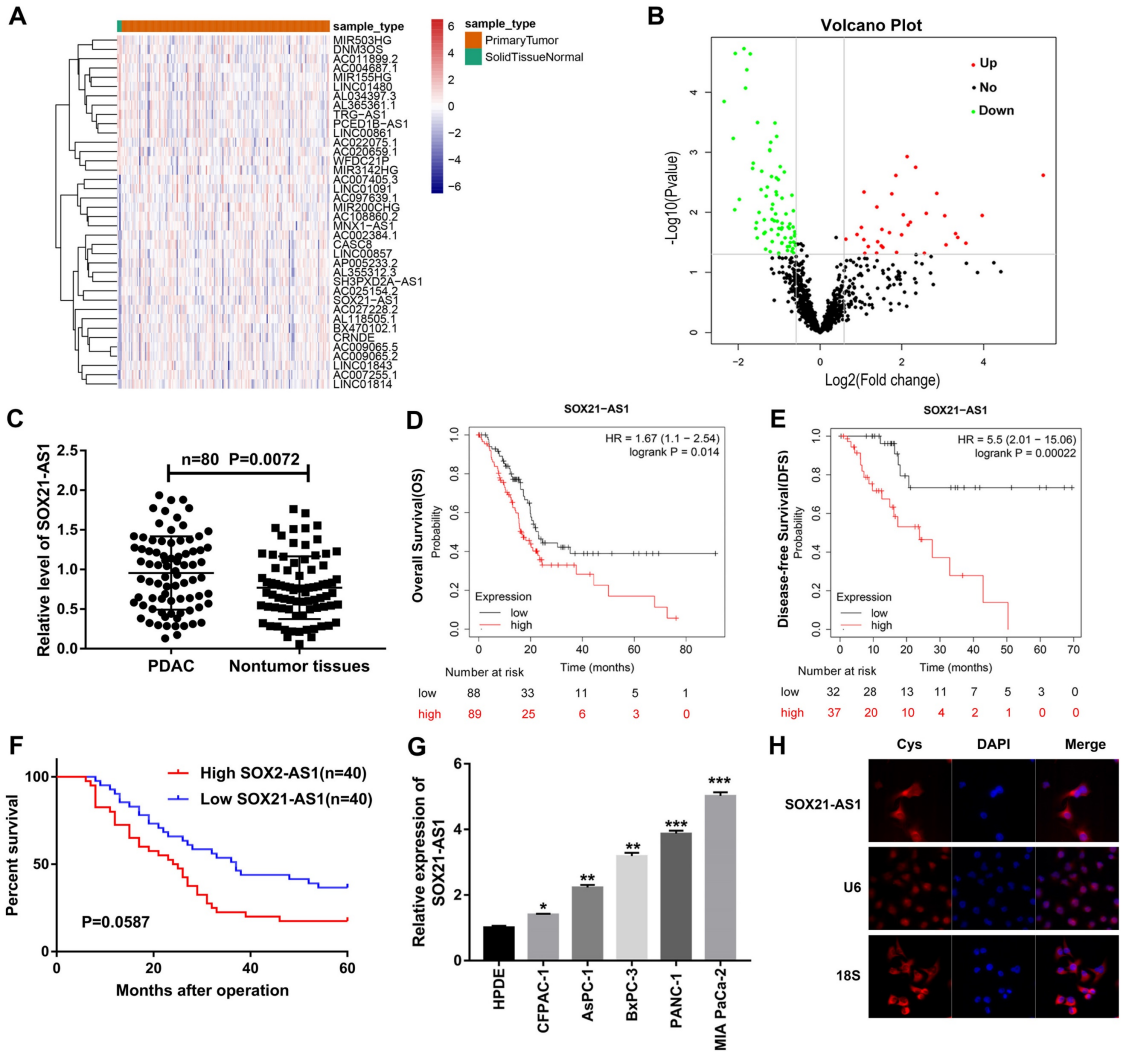
** The expression of SOX21-AS1 was up-regulated in PDAC and was associated with poor prognosis.** A. Heat map from TCGA database. B. Volcanic plot of lncRNAs. C. PCR detection of SOX21-AS1 expression in 80 pairs of PDAC and nontumor tissues. D. Survival curve of SOX21-AS1 (Kaplan-Meier database). E. Disease-free survival curve of SOX21-AS1 (Kaplan-Meier database). F. Survival curve of PDAC patients. G. PCR detection of SOX21-AS1 expression in PDAC cell lines. H. FISH assay was used to detect the subcellular localization of SOX21-AS1 in PANC-1. The p values represent comparisons between groups (*p < 0.05, **p < 0.01, ***p < 0.001).

Based on the TCGA database data, we screened lncRNAs with significant differences in expression between PDAC and nontumor tissues (Fig. 1A, B). We detected the expression difference of lncRNAs in 80 pairs of PDAC and nontumor tissue by RT-qPCR and predicted the correlation between lncRNAs and the prognosis of PDAC from the database. We focused on SOX21-AS1, which was shown to be upregulated in PDAC tissues (Fig. 1C) and negatively correlated with the overall survival (OS) and disease-free survival (DFS) of PDAC patients in the Kaplan-Meier (Fig. 1D, E) and StarBase databases (Fig S1A). In addition, 80 PDAC patients were followed up and analyzed, and the results were consistent with the prediction of the database. The expression of SOX21-AS1 was negatively correlated with the survival rate of PDAC patients (Fig. 1F). The clinicopathological characteristics of PDAC patients were analyzed, and the results indicated that the expression of SOX21-AS1 in PDAC patients was closely related to tumor stage, lymphatic/liver metastasis, tumor location, and vascular and nerve invasion (Table 1). The expression of SOX21-AS1 in PDAC cell lines was also significantly higher than that in normal human pancreatic cells (Fig. 1G). FISH images showed that SOX21-AS1 was distributed in the cytoplasm and nucleus of PDAC cells, and most of them were located in the cytoplasm (Fig. 1H). Based on the above results, we found that the expression of SOX21-AS1 was upregulated in PDAC and correlated with poor prognosis.

### SOX21-AS1 promotes the proliferation, migration, invasion and stemness of PDAC *in vitro*

**Figure 2:**
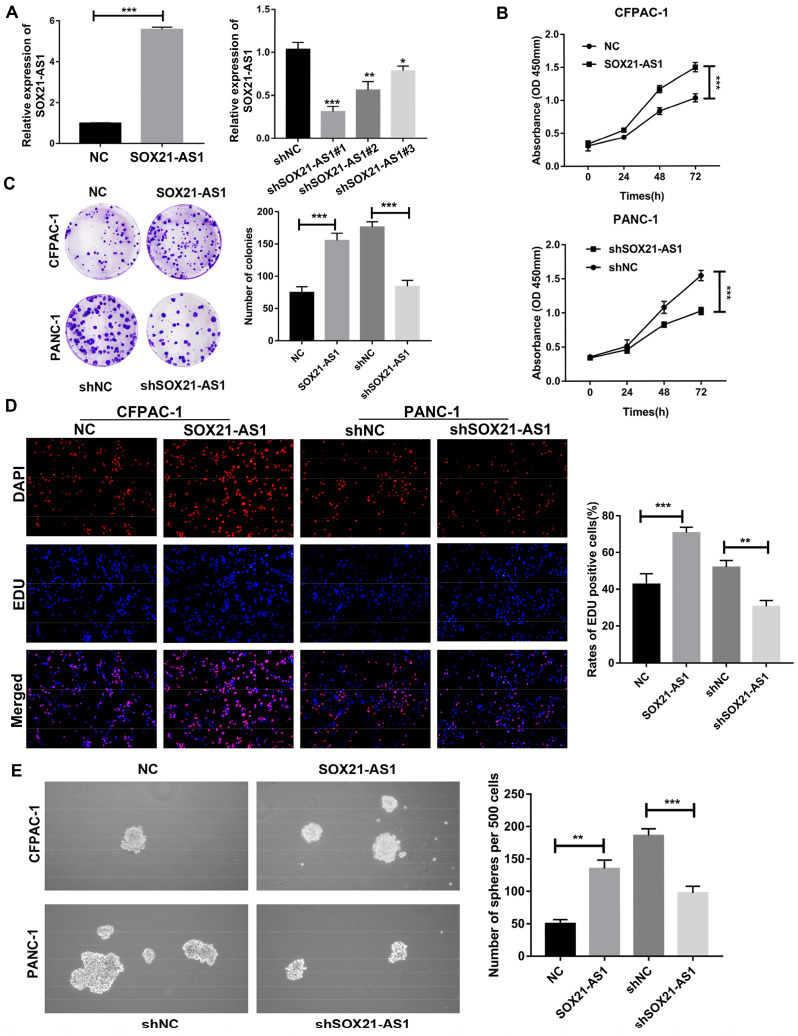
**The expression of SOX21-AS1 is correlated with the proliferation and stemness of PDAC *in vitro*.** A. The expression of SOX21-AS1 in PDAC cells after transfection. B. Growth curves based on CCK-8 assay in transfected cells. C. Representative images of colony formation assay of transfected cells. D. The results of the EdU assay showed that up-regulation and downregulation of SOX21-AS1 in PDAC cells effects cell proliferation. E. The result of tumor sphere-forming assay demonstrated that the stemness of PDAC cells was directly proportional to the expression of SOX21-AS1. The p values represent comparisons between groups (*p < 0.05, **p < 0.01, ***p < 0.001).

Due to the poor tumor-forming ability of MIA PaCa-2 cells in *in vivo* experiments, PANC-1 cells with the second highest expression of SOX21-AS1 were selected for loss-of-function assays, whereas CFPAC-1 cells with the lowest expression were selected for gain-of-function assays, based on the RT-qPCR results of PDAC cells. To determine the role of SOX21-AS1 in PDAC development, we downregulated SOX21-AS1 in PANC-1 cells and upregulated SOX21-AS1 in CFPAC-1 cells by transfection with lentivirus. The efficiency of cell transfection was validated by RT-qPCR (Fig. 2A). Then, we selected shSOX21-AS1#1 cells with higher knockdown efficiency for follow-up assays. CCK-8, colony formation and EdU assays showed that SOX21-AS1 facilitated CFPAC-1 cell proliferation, whereas this effect was partially attenuated by SOX21-AS1 suppression, and the opposite results were observed in PANC-1 cells (Fig. 2B-D). Sphere formation has been used to detect and assess the characteristics of cancer stem cells (CSCs) from PDAC. Cancer stem cells have been proposed to be a driving force in tumor recurrence and metastasis 34. In the sphere formation assay, SOX21-AS1 enhanced the stemness of PDAC cells (Fig. 2E).

**Figure 3:**
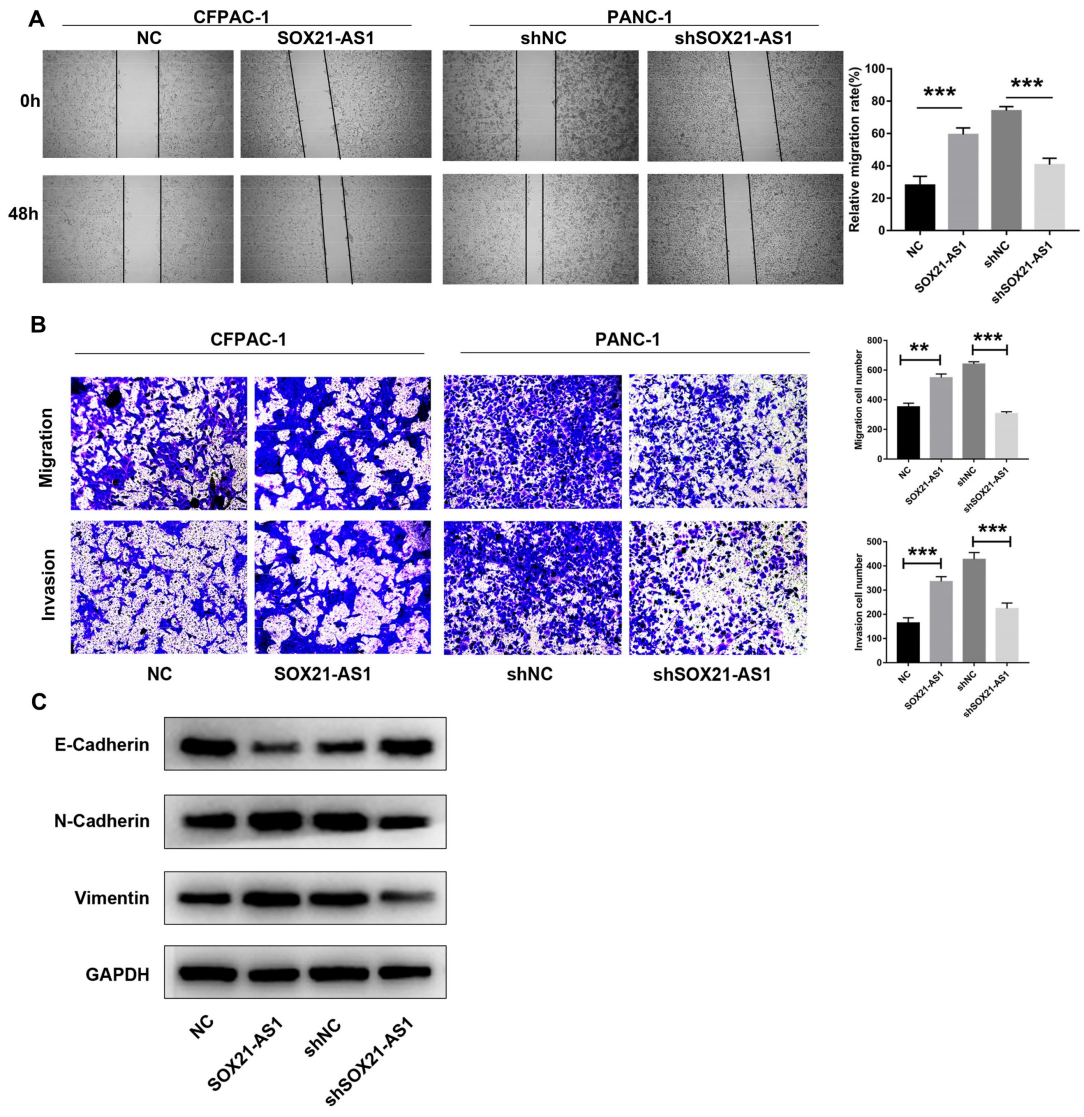
** The expression of SOX21-AS1 is correlated with the migration and invasion of PDAC *in vitro*.** A. Wound healing assay suggested that the up-regulation of SOX21-AS1 elevated the migration of PDAC cell lines. B. Transwell assays presented that the transfected cells changed invasion and migration ability in PDAC. C. E-Cadherin, N- Cadherin and Vimentin protein expression levels of the above transfected cells. The p values represent comparisons between groups (*p < 0.05, **p < 0.01, ***p < 0.001).

The scratch wound healing assay results showed that the migration ability of PDAC cells after transfection was positively proportional to the expression of SOX21-AS1 (Fig. 3A). Furthermore, using transwell experiments, it was found that the migration and invasion ability of CFPAC-1 cells increased with increasing SOX21-AS1 expression, whereas the migration and invasion of PANC-1 cells weakened with decreasing SOX21-AS1 expression (Fig. 3B). Epithelial-mesenchymal transformation (EMT) is an important mechanism that enhances the ability of PDAC cells to metastasize. We detected the expression of EMT-related biomarkers, including E-cadherin, N-cadherin and Vimentin. Further analysis showed that the expression of E-cadherin was significantly decreased in CFPAC-1 cells overexpressing SOX21-AS1, whereas it was increased in PANC-1 cells suppressing SOX21-AS1. However, the expression changes in N-cadherin and Vimentin showed the opposite results in the above cell lines (Fig. 3C). These results showed that SOX21-AS1 promotes proliferation and EMT of PDAC *in vitro*.

### SOX21-AS1 stimulates proliferation of PDAC *in vivo*

**Figure 4:**
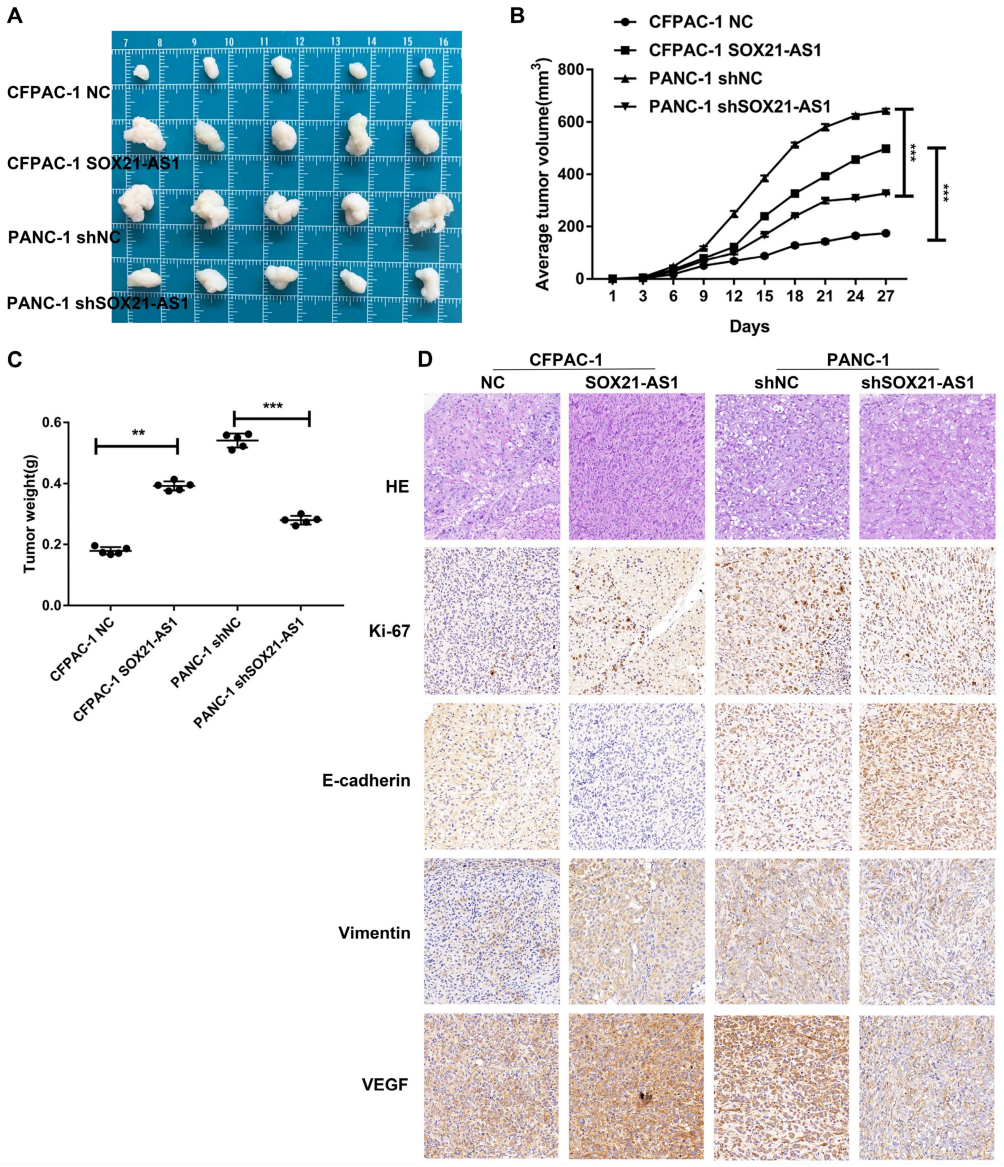
***In vivo* experiments showed that SOX21-AS1 could promote the development of PDAC.** A. Photograph of tumors obtained from nude mice. B. Tumor volume growth curve. C. Tumor weight. D. Representative images of HE staining and Immunohistochemical detection of Ki67, E-cadherin, Vimentin and VEGF protein levels in four groups of subcutaneous tumor tissues (x400). The p values represent comparisons between groups (*p < 0.05, **p < 0.01, ***p < 0.001).

We established a subcutaneous tumor model to further study the carcinogenic effect of SOX21-AS1 *in vivo*. The mice were divided into four groups (CFPAC-1 NC, CFPAC-1 SOX21-AS1, PANC-1 shNC, and PANC-1 shSOX21-AS1) in subcutaneous tumor-forming experiments of nude mice. Overexpression of SOX21-AS1 stimulated subcutaneous tumor growth in nude mice, whereas downregulation of SOX21-AS1 slowed the growth rate of subcutaneous tumors in nude mice; the results were reflected in tumor weight and volume (Fig. 4A-C). Immunohistochemical staining of the xenograft tumor tissue showed that the expression of Ki67 and Vimentin in the CFPAC-1 SOX21-AS1 group was significantly increased compared with that in the CFPAC-1 NC group, whereas the expression of E-cadherin was decreased. In addition, when the expression of SOX21-AS1 was elevated, the expression of VEGF was also increased (Fig. 4D). These data suggest that SOX21-AS1 stimulates the proliferation of PDAC *in vivo*.

### Exosomal SOX21-AS1 secreted by PDAC cells was accepted by HUVECs and promoted angiogenesis

**Figure 5:**
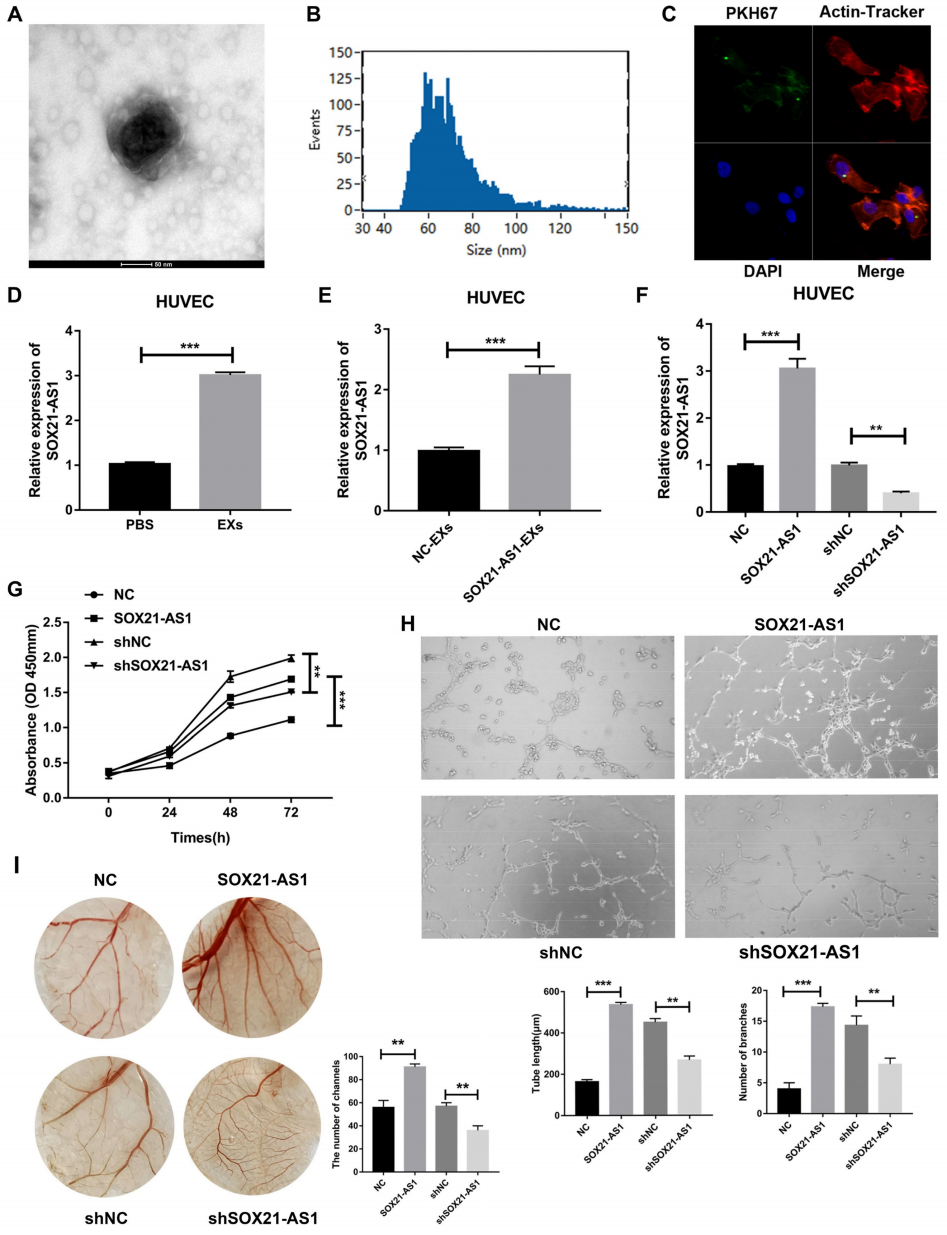
**Exosomal SOX21-AS1 secreted by PDAC promoted angiogenesis of HUVECs.** A. Representative images showing the morphology of EXs by electron microscopy (scale bar, 100 nm). B. Exosomal size, distribution, and concentration measured by nanoparticle tracking analysis. C. Representative images showing endocytosis of PDAC cell-derived exomes via laser scanning confocal microscopy (LSCM; ×400). D. The result of RT-qPCR detected the relative expression level of SOX21-AS1 after co-culture of exosomes. E. RT-qPCR was used to detect the relative expression of SOX21-AS1 after co-culture with exosomes from control and overexpressed group of PANC-1 cells. F. The expression of SOX21-AS1 in HUVEC was knocked down and overexpressed by lentivirus transfection. H. CCK8 assay reflected cell proliferation ability. G. Tube formation assay was used to detect the effect of SOX21-AS1 on angiogenesis *in vitro*. I. CAM assay was used to detect the effect of SOX21-AS1 on angiogenesis *in vivo*. The p values represent comparisons between groups (*p < 0.05, **p < 0.01, ***p < 0.001).

Overexpression of SOX21-AS1 caused overexpression of VEGF in subcutaneous tumor tissues and vice versa. We hypothesized that SOX21-AS1 can be secreted from PDAC to HUVECs by exosomes to advance the angiogenesis of HUVECs. The results of electron microscope analysis nanoparticle tracking analysis (NTA) showed that PANC-1 cell-derived EXs were round with diameters between 30 and 150 nm (Fig. 5A, B). Images taken by confocal microscopy showed that HUVECs could endocytose EXs derived from PANC-1 cells (Fig. 5C). We then treated HUVECs with PANC-1 cell-derived EXs. Compared with that in the PBS treatment group, the expression of SOX21-AS1 of HUVECs was increased in PANC-1 cell-derived EXs group (Fig. 5D). To determine whether SOX21-AS1 affects the angiogenesis of HUVECs, we transfected PANC-1 cells with SOX21-AS1 or NC and cocultured the transfected cells with HUVECs. RT-qPCR was used to detect the expression of SOX21-AS1 in cocultured HUVECs. The results showed that the expression of SOX21-AS1 was significantly elevated in HUVECs cocultured with PANC-1 SOX21-AS1 cells compared with that of the PANC-1 NC group (Fig. 5E). HUVECs could change the expression of SOX21-AS1 through PANC-1 cell-derived EXs. SOX21-AS1 in HUVECs was knocked down or overexpressed by lentivirus transfection (Fig. 5F). Subsequently, the CCK8 assay showed that SOX21-AS1 could promote the proliferation of HUVECs (Fig. 5G). Next, we conducted tube formation assays (*in vitro*) and CAM assays (*in vivo*) to research the effects of SOX21-AS1 on angiogenesis. The results showed that overexpression of SOX21-AS1 increased the angiogenesis of HUVECs and vice versa (Fig. 5H, I). The exosomal SOX21-AS1 secreted by PDAC promotes the proliferation and angiogenesis of HUVECs.

### SOX21-AS1 competitively inhibited miR-451a expression in PDAC

**Figure 6:**
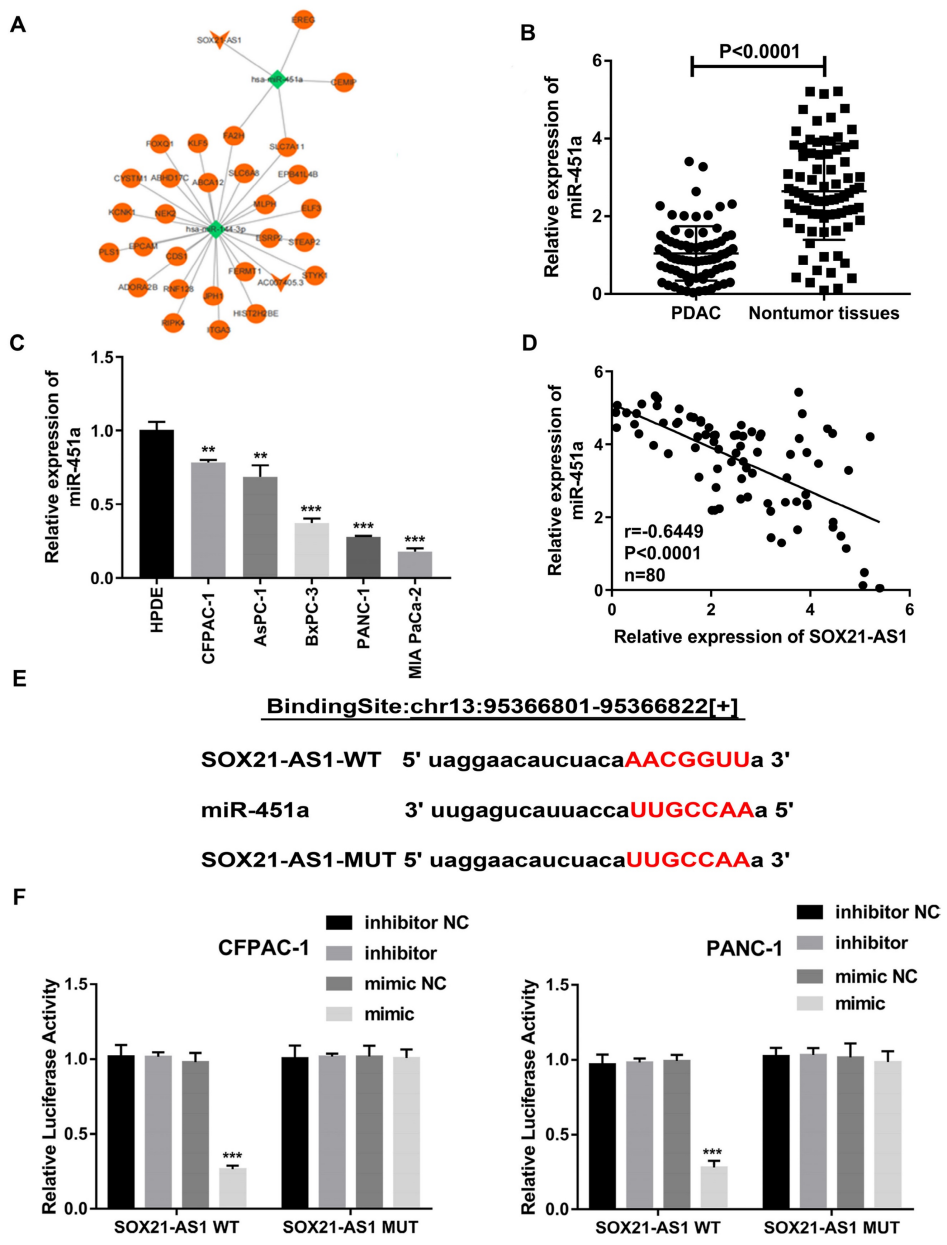
**Searching for the downstream miRNA of SOX21-AS1.** A. TCGA database bioinformatics network analysis diagram. B. The expression levels of miR-451a in PDAC and nontumor tissues. C. Expression of miR-451a in PDAC cell lines. D. Correlation analysis of SOX21-AS1 and miR-451a in PDAC tissue. E. The predicted miR-451a targeting sequence with SOX21-AS1. F. Luciferase activity was detected through luciferase reporter assay. The p values represent comparisons between groups (*p < 0.05, **p < 0.01, ***p < 0.001).

According to the results of the FISH experiment, SOX21-AS1 was located in both the nucleus and cytoplasm. SOX21-AS1 located in the cytoplasm could be used as a ceRNA, a common mechanism of posttranscriptional regulation mediated by lncRNAs to sponge miRNAs. We further studied the potential molecular biological mechanism of SOX21-AS1 in PDAC. We constructed a possible ceRNA regulatory network for SOX21-AS1 through the TCGA database. The results showed that miR-451a might be a potential downstream target of SOX21-AS1 and its four potential downstream target genes (Fig. 6A). As predicted by the StarBase database, the expression of miR451a was decreased in PDAC (Fig S1B). The prediction results of the StarBase database also showed that the expression of miR-451a in PDAC was negatively correlated with the expression of SOX21-AS1 (Fig S1C). We used RT-qPCR to detect the expression of miR-451a in 80 pairs of PDAC tissues and related adjacent nontumor tissues. The expression of miR-451a was downregulated in PDAC tissues and cell lines (Fig. 6B, C). A Spearman correlation analysis showed that SOX21-AS1 expression was negatively correlated with miR-451a (Fig. 6D). We then performed a dual-luciferase reporter assay to verify that SOX21-AS1 directly binds to miR-451a and confirmed this hypothesis (Fig. 6E). Wild-type (WT) or mutant (MUT) SOX21-AS1 3'UTR was inserted into the reporter vector. The results of the dual-luciferase reporter assay showed that the relative luciferase activity of CFPAC-1 and PANC-1 cells cotransfected with miR-451a and wild-type SOX21-AS1 was significantly decreased compared with those of cells transfected with mutants (MUT-SOX21-AS1) (Fig. 6F). Therefore, we found that SOX21-AS1 directly binds to miR-451a in PDAC.

### EREG is a target gene of miR-451a

**Figure 7:**
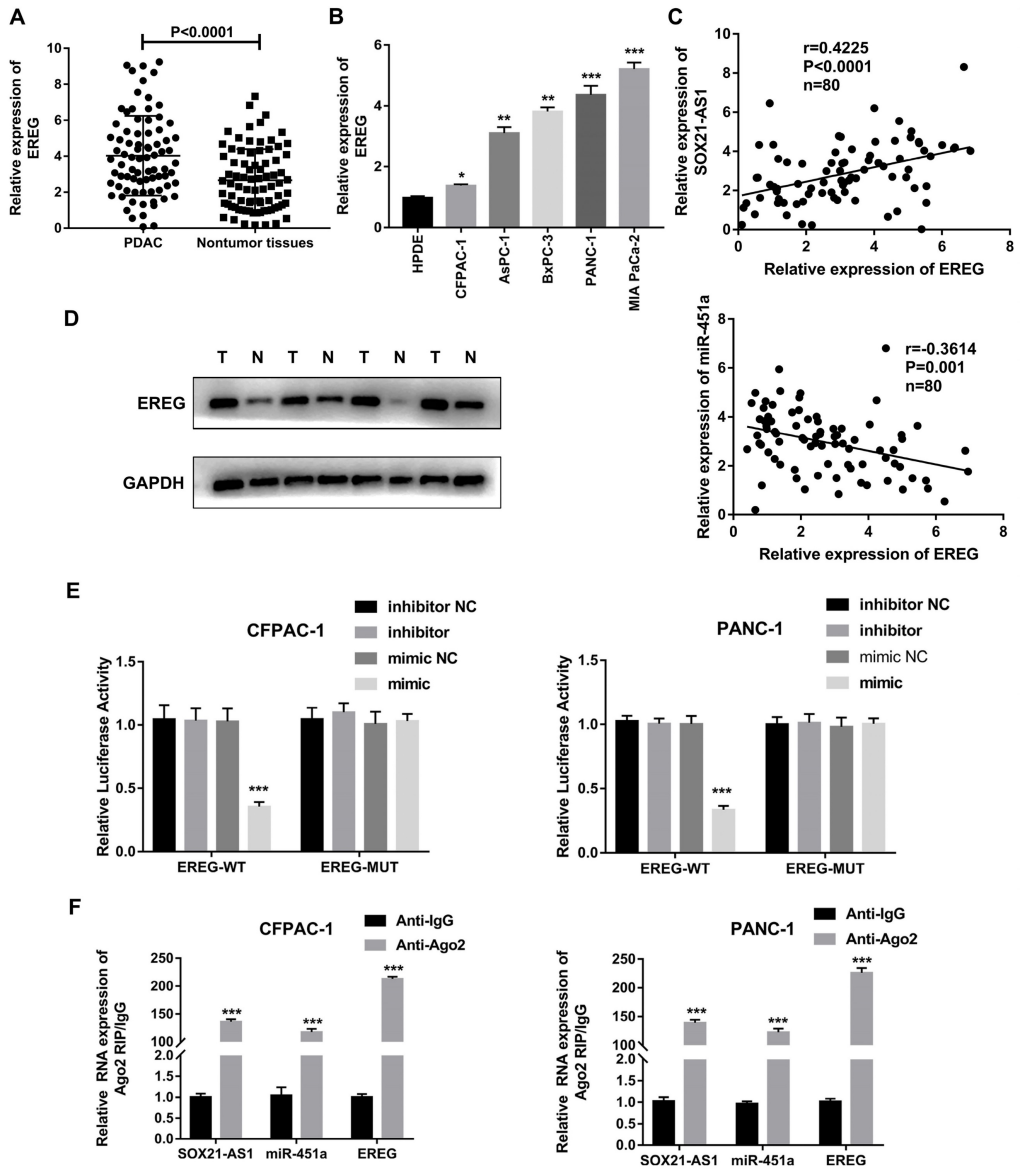
**EREG is the downstream target gene of SOX21-AS1 and miR-451 in PDAC.** A. The expression mRNA levels of EREG in PDAC. B. The expression of EREG in PDAC cell lines. C. In PDAC tissue, the expression of EREG was directly proportional to SOX21-AS1 and inversely proportional to miR-451a. D. The protein levels of EREG in PDAC samples were detected by western blot (T: tumor, N: nontumor). E. luciferase activity was detected in luciferase reporter assay. F. RIP assay was used to detect the binding of SOX21-AS1/miR-451a/EREG axis in PDAC cell lines. The p values represent comparisons between groups (*p < 0.05, **p < 0.01, ***p < 0.001).

According to the above network diagram from the TCGA database, we found that there are four possible downstream target genes for miR-451a. The StarBase databases predicted that the expression of EREG was elevated in PDAC (Fig S2A). According to the StarBase, GEPIA and Kaplan-Meier databases, only EREG was predicted to be associated with poor prognosis for PDAC (Fig S2B-D). EREG was highly expressed in PDAC tissues and cell lines (Fig. 7A, B). A Spearman correlation analysis showed that the expression of EREG was negatively correlated with the expression of miR-451a and positively correlated with the expression of SOX21-AS1 (Fig. 7C). In addition, EREG protein levels in PDAC tissues were also higher than those in related adjacent nontumor tissues (Fig. 7D). We verified the interaction between miR-451a and EREG with a dual-luciferase reporter assay, and the results confirmed that miR-451a and EREG could bind directly (Fig. 7E). To further study the relationship among SOX21-AS1, miR-451a and EREG in PDAC, we conducted a RIP assay, and the results showed that SOX21-AS1, miR-451a and EREG were significantly enriched in the anti-AGO2 group compared with the anti-IgG negative control group (Fig. 7F). Therefore, we hypothesized that SOX21-AS1 could affect the downstream target gene EREG through competitive binding with miR-451a.

### The SOX21-AS1/miR-451a/EREG axis promotes the malignant ability of PDAC

**Figure 8:**
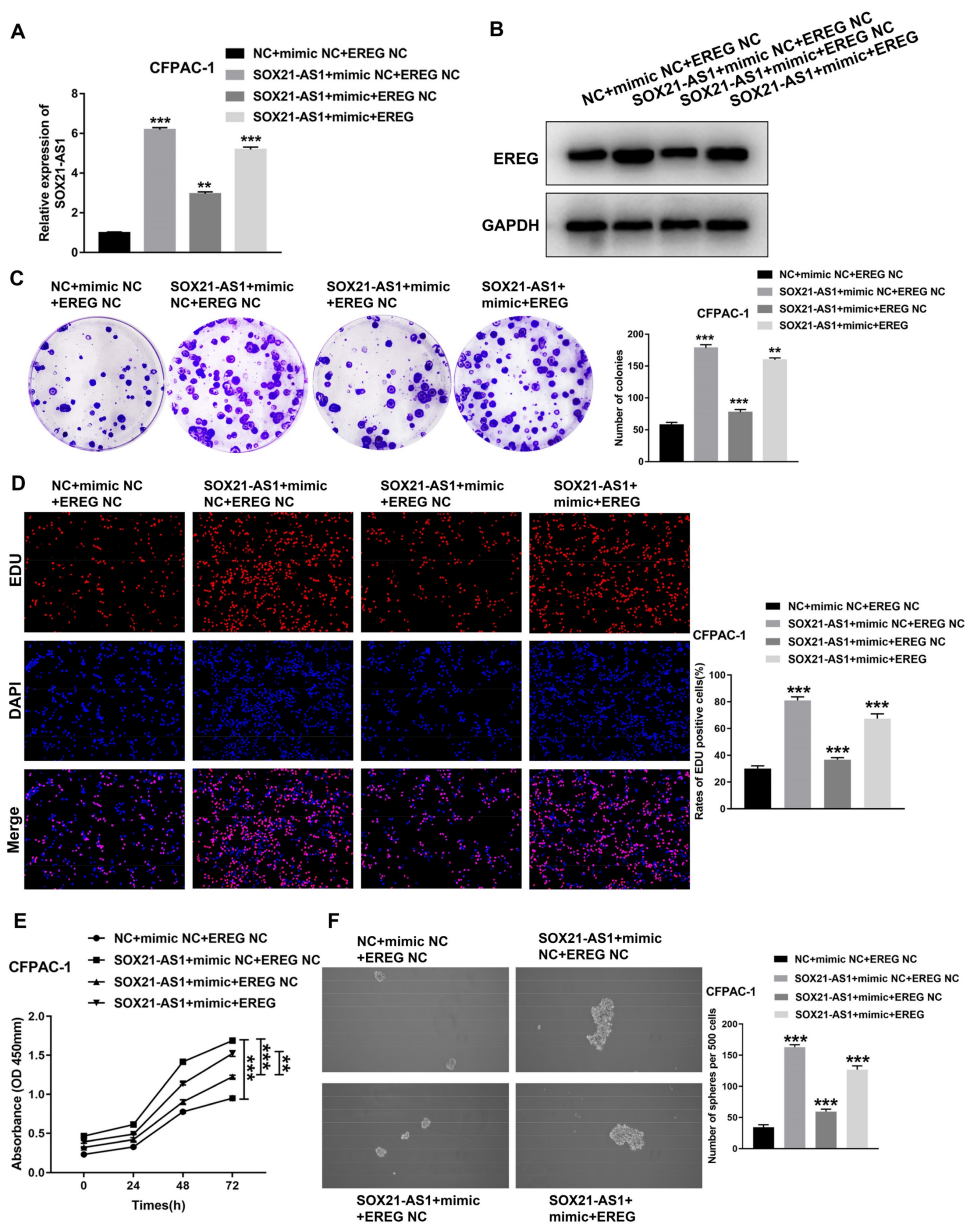
**Regulation of proliferation and stemness of tumor cells by SOX21-AS1/miR451a/EREG axis in PDAC.** A. The expression of SOX21-AS1 in transfected cells. B. The expression of EREG in transfected cells by western blot. C. Representative images of colony formation assay of transfected cells. D. The results of the EdU assay. E. Growth curves based on CCK-8 assay in transfected cells. F. The result of tumor sphere-forming assay. The p values represent comparisons between groups (*p < 0.05, **p < 0.01, ***p < 0.001).

**Figure 9:**
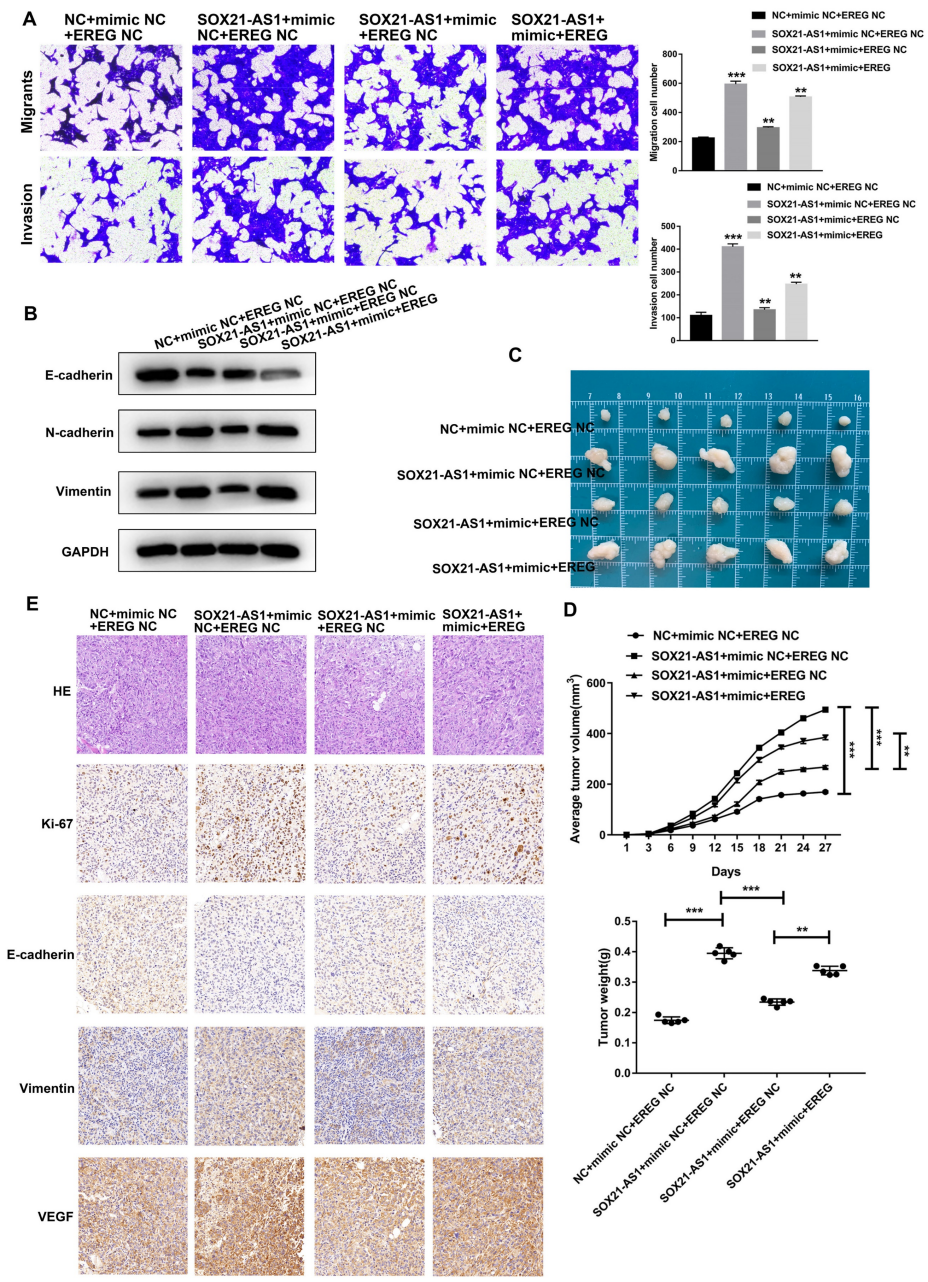
**SOX21-AS1/miR451a/EREG axis regulated the proliferation, migration and invasion of PDAC, and was related to EMT.** A. Cell migration and invasion ability of PDAC cells were evaluated by transwell assays. B. Detection of EMT-related proteins by western blot. C. Photograph of tumors obtained from nude mice. D. Tumor volume and weight were recorded and analyzed. E. Representative images of HE staining and Immunohistochemical detection of subcutaneous tumor tissues (x400). The p values represent comparisons between groups (*p < 0.05, **p < 0.01, ***p < 0.001).

To further investigate the role of the axis in PDAC, we conducted a series of functional recovery experiments. CFPAC-1 cells were treated with NC+mimic NC+EREG NC, SOX21-AS1+mimic NC+EREG NC, SOX21-AS1+mimic+EREG NC or SOX21-AS1+mimic+EREG. The transfection efficiency was verified by RT-qPCR assays (Fig. 8A). The western blot assay results confirmed the expression of EREG in transfected cells (Fig. 8B). The mimic could rescue the upregulated effect of SOX21-AS1 on the proliferation and stemness of PDAC cells. The upregulation of EREG also reversed the rescue effect of the mimic (Fig. 8C-F). The mimic and EREG also played a similar role in the migration and invasion of PDAC (Fig. 9A). In addition, the downregulation of EMT-related proteins (N-cadherin and Vimentin) and upregulation of E-cadherin by miR-451a were also reversed by EREG (Fig. 9B). *In vivo* experiments also further verified that miR-451a and EREG could reverse the regulation of SOX21-AS1 in PDAC (Fig. 9C, D). The immunohistochemical results suggested that the regulation of the SOX21-AS1/miR-451a/EREG axis in PDAC was related to the expression of Ki67, EMT and VEGF (Fig. 9E). We can conclude that the SOX21-AS1/miR-451a/EREG axis promotes the malignant ability of PDAC.

## Discussion

Pancreatic cancer is an aggressive form of gastrointestinal cancer. The most common pathologic type is pancreatic ductal adenocarcinoma (PDAC) 35. lncRNAs are considered to play a central role in the pathogenesis and biology of PDAC, affecting tumor growth, migration and invasion by regulating cell processes such as epithelial-mesenchymal transformation, and lncRNAs may become biomarkers for diagnosis, prognosis and prediction 36. For example, lncRNA MALAT1 is highly expressed in PDAC tissues and associated with chemotherapy resistance. In addition, it also binds to HuR to modulate TIA-1-mediated autophagy activation at the posttranscriptional level. It can also regulate KRAS expression and promote the proliferation of PDAC cells through competitive inhibition 37, 38. Using the TCGA database, we screened out lncRNAs with significant differences in expression between PDAC tissues and paracancerous tissues. Combined with other bioinformatics analysis databases, we selected SOX21-AS1 as the target lncRNA of our research. We found that SOX21-AS1 was highly expressed in PDAC tissues and cells and was associated with poor prognosis. The results of *in vivo* and *in vitro* assays confirmed that SOX21-AS1 promoted the proliferation, migration, invasion and stemness of PDAC cells. LncRNA plays an important regulatory role in the occurrence and development of PDAC, among which the expression of SOX21-AS1 is increased in PDAC and is associated with poor prognosis. At the same time, SOX21-AS1 promotes the malignant biological behavior of PDAC.

The mechanism underlying the role of lncRNAs in tumors is determined by their subcellular localization. After lncRNAs are produced in the nucleus, they can interact with nuclear matrix proteins to localize to the nucleus and regulate downstream genes 11. For example, the lncRNA HOTTIP can directly bind to the WDR5 protein to activate HOXA gene transcription 39, 40. lncRNAs can also be exported to the cytoplasm to play a regulatory role. There is increasing evidence that cytoplasmic lncRNAs can act as decoys for miRNAs and proteins, thereby affecting gene regulation. It has been reported that LINC-MD1 binds to miR-133 to activate muscle-specific gene expression 41. The lncRNA NORAD regulates the stability and translation of PUMILIO-bound mRNA by inhibiting the PUMILIO protein 42. Using FISH assays, we found that SOX21-AS1 was mainly distributed in cytoplasm of PDAC. Based on previous research results, we hypothesized that SOX21-AS1 located in the cytoplasm regulates PDAC by acting as a ceRNA to adsorb miRNA. Furthermore, we constructed a SOX21-AS1-related lncRNA-miRNA-mRNA network through the TCGA database. Combined with other bioinformatics-related databases and experimental data *in vitro* and *in vivo*, we verified that SOX21-AS1 in the cytoplasm can regulate EREG expression by binding miR-451a, thus promoting the proliferation, migration, invasion and stemness of PDAC. SOX21-AS1 regulates the expression of EREG by binding to miR-451a to accelerate the development of PDAC.

Extensive fibrosis and low vascularization are the main features of PDAC 43. Angiogenesis can enhance the proliferation of PDAC 44. Angiogenesis-related factors can also be therapeutic targets for PDAC. Existing studies have shown that antiangiogenic therapy can effectively inhibit tumor growth in animal models of PDAC. The antiangiogenic agent TNP-470 can reduce angiogenesis, tumor growth and metastasis in pancreatic cancer cell lines 45. In cancer, exosomes (EXs) may be involved in tumor growth and metastasis by regulating the immune response, epithelial-mesenchymal transformation and angiogenesis and can also be used as noninvasive biomarkers for the early detection and diagnosis of cancer. Due to their amphiphilic structure, EXs are also a natural drug carrier for cancer treatment 46. EXs secreted by PDAC cells transport proteins, mRNAs, lncRNAs and miRNAs between cells and can affect the tumor microenvironment 47. Tumor-derived lncRNAs can be delivered into endothelial cells via exosomes to promote angiogenesis 48. Exosomal lncRNA-H19 promotes osteogenesis and angiogenesis by mediating Angpt1/Tie2-NO signaling in CBS heterozygous mice 49. Glioma cells promote angiogenesis by releasing exosomal lncRNA POU3F3 50. Although SOX21-AS1 plays an oncogenic role in a variety of tumors such as lung cancer, breast cancer, cervical cancer, and colorectal cancer 51. However, SOX21-AS1 has not been studied in exosomes. Meanwhile, the study of Kamerkar *et al.* provided valid evidence for the role of exosomes in the treatment of PDAC 52. In this study, we first found the presence of SOX21-AS1 in PDAC exosomes. And the experimental results of tube formation and CAM assays proved that exosomal SOX21-AS1 from PDAC cells promotes the angiogenesis of PDAC. Exosomal SOX21-AS1 promotes the angiogenic ability of PDAC.

Due to the spatial and temporal heterogeneity of tumors, traditional biopsy methods cannot obtain complete and dynamic tumor tissue information. In recent years, in order to overcome the shortcomings of traditional tissue biopsy, a new diagnostic technique called "liquid biopsy" has been developed 53. Studies have shown that lncRNAs can stably exist in the blood of patients through exosomes secreted by tumor cells, which may provide novel biomarkers for clinical diagnosis and prognostic evaluation of patients 54. This study provides a possibility for SOX21-AS1 in exosomes to become a marker for the diagnosis and treatment of PDAC and a prognostic predictor for patients.

## Conclusion

In summary, our results showed that SOX21-AS1 was upregulated in PDAC and accelerated cell proliferation, migration, invasion, stemness and EMT progression through the SOX21-AS1/miR-451a/EREG axis. In addition, exosomal SOX21-AS1 secreted by PDAC promoted HUVECs angiogenesis. This discovery may provide novel targets for the early diagnosis and therapy of PDAC.

## Supplementary Material

Supplementary figures and tables.

## Figures and Tables

**Table 1:** The relationship between SOX21-AS1 expression level in PDAC tissues and clinical parameters of patients.

Clinicopathological features		All cases	SOX21-AS1		P value
			High expression	Low expression	
Age (years)	>65	46	24	22	0.6511
	≤65	34	16	18	
Gender	Male	33	17	16	0.8203
	Female	47	23	24	
Tumor stage*	Stage I-II	47	18	29	0.0125
	Stage III-IV	33	22	11	
Lymphatic/liver metastasis*	Positive	34	25	9	0.0003
	Negative	46	15	31	
Tumor location*	Head of pancreas	47	28	19	0.0410
	Body of pancreas	33	12	21	
Vascular invasion*	Positive	48	29	19	0.0225
	Negative	32	11	21	
Nerve invasion*	Positive	52	31	21	0.0191
	Negative	28	9	19	
