# Supplementary material for: Exosomal SOX21-AS1 Regulates EREG by Sponging miR-451a and Promotes the Malignancy of Pancreatic Ductal Adenocarcinoma
Source: J Cancer. 2024 Apr 23;15(11):3321–37. doi: 10.7150/jca.95014 (PMC11134441; doi:10.7150/jca.95014)
Supplement: Supplementary file 1 — Supplementary figures and tables. [file jcav15p3321s1.pdf]

# Supplementary material

## Table S1 Quantitative PCR primers and corresponding sequences

| Gene | Forward (5'–3') | Reverse (5'–3') |
|---|---|---|
| SOX21-AS1 | AGCTACGGAGGAAGAGGGTT | TCAGCAGCGCATGTAAGTGA |
| miR-451a | GCGCAAACCGTTACCATTAC | GTGCAGGGTCCGAGGT |
| EREG | CACCGAGAGAAGGATGGAGA | GTGTCCATGCAAGCAGTAGC |
| GAPDH | TCGACAGTCAGCCGCATCTTCTTT | ACCAAATCCGTTGACTCCGACCTT |
| U6 | CTCGCTTCGGCAGCACA | AACGCTTCACGAATTTGCGT |

## Table S2 Antibodies used in the present study

| Protein | WB | IHC | Product code |
|---|---|---|---|
| EREG | 1:1000 | | Cell Signaling Technology, #12048 |
| Ki67 | | 1:200 | Abcam, ab16667 |
| E-cadherin | 1:1000 | 1:400 | Cell Signaling Technology, #3195 |
| N-cadherin | 1:1000 | 1:400 | Cell Signaling Technology, #13116 |
| Vimentin | 1:1000 | 1:200 | Cell Signaling Technology, #5741 |
| GAPDH | 1:1000 | | Cell Signaling Technology, #5174 |
| VEGF | | 1:200 | Abcam, ab32152 |

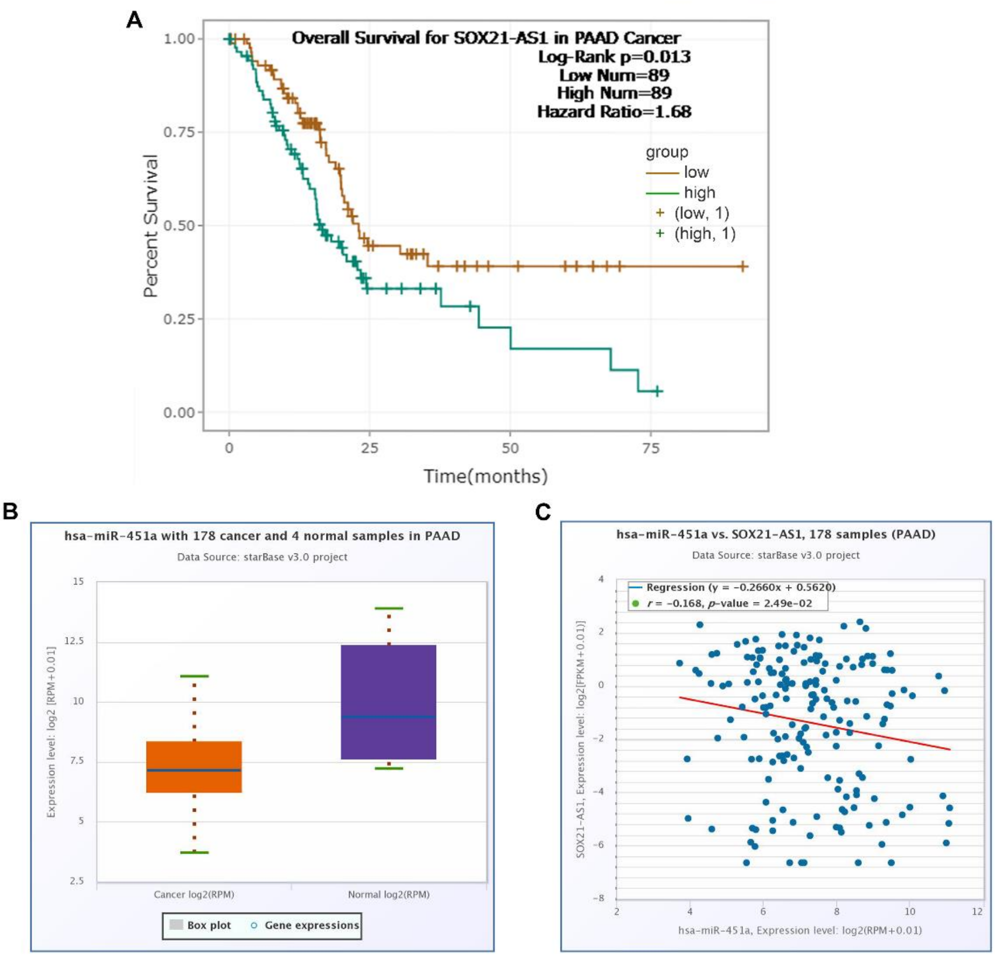


**Fig S1.** Bioinformatics database predicted the data of SOX21-AS1/miR451a/EREG axis in PDAC. A. The StarBase database predicted that SOX21-AS1 was associated with poor outcomes in patients with PDAC. B. Predicted results of the expression of miR-451a in PDAC. C. Correlation analysis data of of SOX21-AS1 and miR-451a in the database.

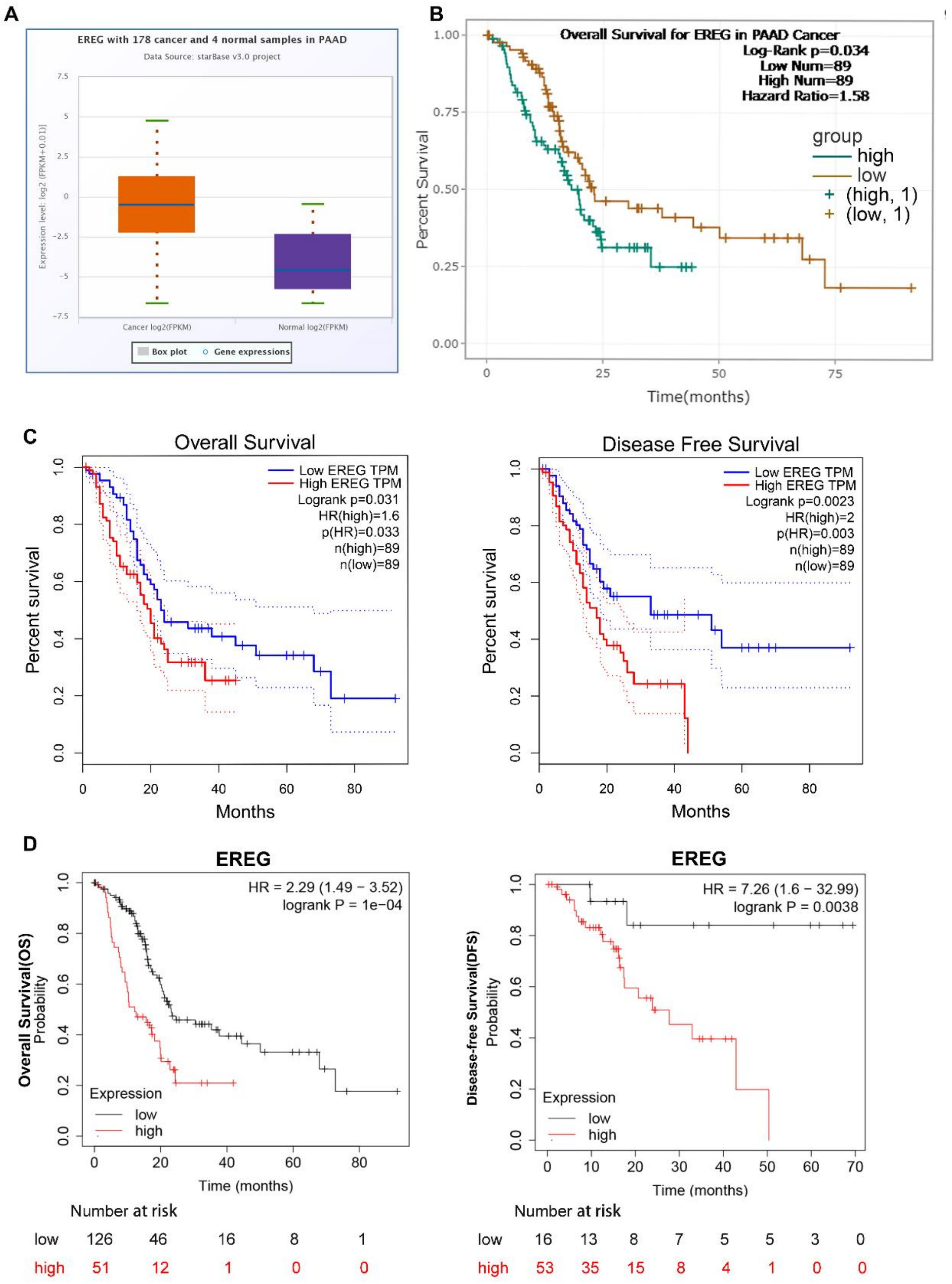


**Fig S2.** Bioinformatics database predicted the data of SOX21-AS1/miR451a/EREG axis in PDAC. A. The expression of EREG in PDAC and normal tissues provided by the StarBase database. B, C and D. The StarBase, GEPIA and Kaplan-Meier databases shown the outcomes of PDAC patients with different EREG expression.
